# Molecular Survey on Vector-Borne Pathogens in Alpine Wild Carnivorans

**DOI:** 10.3389/fvets.2020.00001

**Published:** 2020-01-23

**Authors:** Elena Battisti, Stefania Zanet, Sara Khalili, Anna Trisciuoglio, Beatrice Hertel, Ezio Ferroglio

**Affiliations:** ^1^Department of Veterinary Science, University of Turin, Turin, Italy; ^2^Department of Parasitology, Faculty of Veterinary Medicine, University of Tehran, Tehran, Iran; ^3^Department of Agricultural, Forest and Food Science, University of Turin, Turin, Italy

**Keywords:** *Babesia*, *Hepatozoon*, carnivores, *Leishmania*, zoonoses, wildlife, vector-borne pathogens

## Abstract

In Europe, free-ranging wildlife has been linked to the emergence of several vector-borne diseases such as rodents for *Borrelia burgdorferi* s.l. In particular, wild carnivorans are one of the most important sources of emerging zoonotic pathogens worldwide, although little information is available regarding the epidemiology of vector-borne parasites in these animals. Thus, the aim of this paper was to investigate the prevalence of *Babesia* spp., *Anaplasma* spp., *Ehrlichia* spp., *Hepatozoon* spp. and *Leishmania infantum* in alpine wild canids and mustelids from Italy. For this study, spleen samples of 157 foxes (*Vulpes vulpes*), 45 badgers (*Meles meles*), and 33 wolves (*Canis lupus*) collected between 2009 and 2017 in Northwest Italy were examined by using conventional PCR. Logistic regression was used to identify possible risk factors for pathogen infections. DNA of any of the tested pathogens was found in more than 90% of the analyzed animals. In particular, *Babesia* spp. showed significantly higher prevalence in foxes (89.7%) and badgers (89.6%) than in wolves, while the latter were considerably more infected with *Hepatozoon canis* (75.8%) than foxes (5.1%). None of the badger tested positive for *Hepatozoon* spp., although they showed high prevalence of *Leishmania infantum* (53.3%). Sequencing results revealed the presence, among others, of *Babesia vulpes, Babesia* sp. isolate badger type A and B, and *Anaplasma phagocytophilum*. Moreover, previously unreported pathogen/host associations were observed, such as *Babesia capreoli* in wolves and badgers. The prevalence of vector-borne pathogens observed in the present study is one of the highest reported so far, suggesting the importance of free-ranging carnivorans in the epidemiology and maintenance of the sylvatic cycle of the pathogens. Moreover, several of these pathogens are of particular importance regarding human (*A. phagocytophilum, L. infantum*) and pet health (*L. infantum, B. vulpes*).

## Introduction

More than 70% of zoonotic emerging infectious diseases are caused by pathogens with a wildlife origin, and their impact on human health is increasing (1). In particular, the increased interactions between humans, domestic animals and wildlife, resulting from human population growth, increase of peri-urban sylvatic animals and habitat fragmentation, have been proposed as a leading cause of pathogen emergence (2).

Carnivorans are well-adapted to urban and peri-urban environments, and among the most important sources of zoonotic pathogens such as rabies (3, 4). In Europe, sylvatic carnivorans as the red fox (*Vulpes vulpes*) and the European badger (*Meles meles*) are widely distributed across the continent, with stable or growing populations in urban and suburban areas (5). Furthermore, in recent years we assisted to the recovery of large carnivores in Europe such as the wolf (*Canis lupus*), which has shown to adapt well to human-dominated landscape (6).

The increase and urbanization of wildlife populations is expected to influence the epidemiology of zoonotic pathogens, as those transmitted by vectors (7). Tick-borne protozoa of the genus *Babesia* are known to infect both domestic and sylvatic carnivores worldwide (8, 9). In Europe, *B. canis* mainly infects dogs, although it has been reported also in the wolf (10) and the red fox (11). Moreover, foxes have been proposed as the natural hosts of *B. vulpes* due to their high infection rate detected and the absence of clinical signs in most of the cases (12). Several genotypes of *Babesia* spp., phylogenetically related to *B. microti*, have been observed in the European badger, such as *Babesia* sp. Meles-HU 1 and *Babesia* sp. badger type A and B (13, 14). Similar to *Babesia*, protozoa of the genus *Hepatozoon* and bacteria of the genera *Anaplasma* and *Ehrlichia* are known to infect domestic and sylvatic mammals, with *H. canis, A. phagocytophilum, A. platys* and *E. canis* having a considerable impact on carnivores (15, 16). Among wildlife, red fox is the most investigated species, showing up to 16 and 90% of prevalence for *A. phagocytophilum* (17) and *H. canis* (18), respectively. Domestic dogs are the main reservoir host for *Leishmania infantum*, a sand-fly transmitted pathogen, although investigations of a recent human leishmaniasis outbreak in Spain have demonstrated the essential role of the Iberian hare (*Lepus granatensis*) in the maintenance of the sylvatic cycle of the parasite (19). Other studies have focused on the detection of *L. infantum* in wild canids, due to their close phylogenetic relationship with dogs, although their role in the epidemiology of the parasite has not been fully understood (20).

In Italy, scant information regarding the prevalence of vector-borne pathogens in sylvatic carnivorans exist, mainly in the red fox (21–23). Thus, the aim was to investigate the occurrence of selected vector-borne pathogens (*Babesia* spp., *Hepatozoon* spp., *Anaplasma* spp., *Ehrlichia* spp. and *L. infantum*) in free-ranging canids and mustelids in the alpine area of Northwest Italy. Notably, these pathogens were chosen due to their significant impact on human and/or animal health, and for their emergence in the studied area (24, 25).

## Materials and Methods

### Study Area

With more than 25,000 km^2^ of extension, Piedmont region (Northwestern Italy) is one of the widest regions in Italy. Its territory is predominantly mountainous and hilly, with plains mainly distributed in the southern and eastern parts of the region. At least a third of the land is covered by forest and natural areas, of which 193,000 ha are protected. It is also highly populated, with a mean density of 172 inhabitants/km^2^ that reach over 6,000 inhabitants/km^2^ in some urban areas. The sampling area ranged from low-urbanized high mountains (up to 1,800 m a.s.l.) to highly-urbanized plains below 300 m a.s.l.

### Sampling

**Figure 1:**
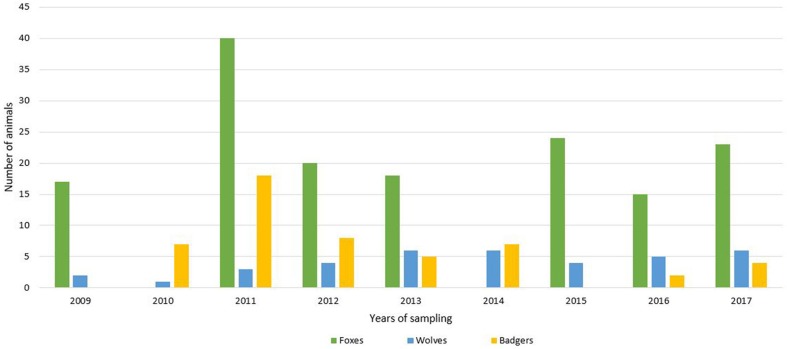
Number of analyzed animals (green = foxes; blue = wolves; yellow = badgers) divided by years of sampling.

For this study, 235 wild carnivorans (157 foxes, 45 badgers and 33 wolves) were collected in the period between 2009 and 2017 (Figure 1). All the animals were road-killed, with the exception of red foxes that were culled during the official hunting seasons as part of the culling program for fox population control, and carcasses were brought to the Department of Veterinary Science, University of Turin, for necropsy.

For each animal, information such as age (estimated by dental conditions and body size measurements), sex and area of origin (mountain/flat/hill region) were recorded (Table 1). Spleen was collected from each animal and individually stored at −20°C until further analysis.

**Table 1:** Analyzed species divided by age and sex.

**Species**	**Age**	**No of animals**	**Sex**	**No of animals**
Red fox (*Vulpes vulpes*)	<1 year	2	M	1
			F	1
	1–2 years	53	M	13
			F	40
	>2 years	102	M	47
			F	55
Wolf (*Canis lupus*)	<1 year	9	M	2
			F	7
	1–2 years	13	M	6
			F	7
	>2 years	11	M	9
			F	2
Badger (*Meles meles*)	<1 year	0	M	0
			F	0
	1–2 years	14	M	12
			F	8
	>2 years	31	M	11
			F	14
Total				235

**Figure 2:**
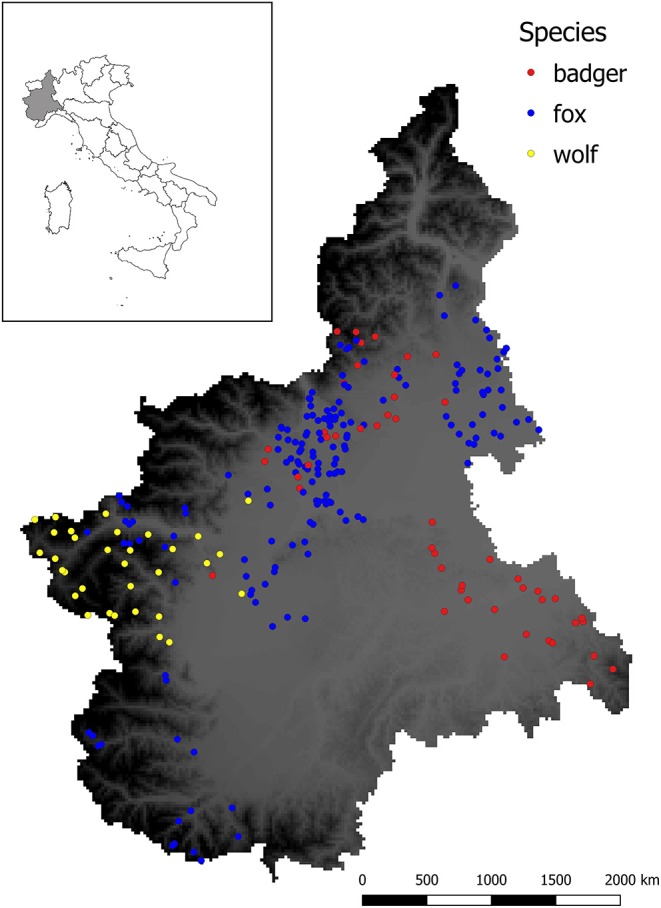
Sampling sites for sylvatic animals analyzed in this study. Dots represent the location in which animals were culled or found dead, whereas different colors indicate the species (red = badger, blue = fox, yellow = wolf). Background color ranging from gray to dark represent the elevation (meters above the sea level m.a.s.l.) of the studied area, with darker color indicating higher elevation.

Figure 2 shows the spatial distribution of the sampled animals.

### Molecular and Statistical Analysis

DNA was extracted from ≈10 mg of tissue by using the commercial kit GenElute Mammalian Genome DNA Miniprep (Sigma-Aldrich, St. Louis, MO, USA).

For *Babesia* detection, a semi-nested PCR targeting the V4 hypervariable region of the 18S rRNA using primers RLB-F2 (5′-GACACAGGGAGGTAGTGACAAG-3′), RLB-R2 (5′-CTAAGAATTTCACCTCTGACAGT-3′) and RLB-FINT (5′-GACAAGAAATAACAATACRGGGC-3′) was performed (21). Briefly, the reaction mixture for the first step contained 1X of PCR Master Mix (Promega Corporation, WI, USA), 20 pmol of each primer and 5 μl of DNA, in an overall volume of 25 μl. The thermal cycler conditions used were an initial denaturation step at 95°C for 5 min, followed by 25 cycles at 95°C for 30 s, 50°C for 45 s and 72°C for 90 s, and a final elongation step at 72°C for 10 min. Amplicons from the first step (1 μl) were used as templates for the second step with internal primer RLB-FINT instead of RLB-F2. Protocol and thermal cycler conditions were identical to the first step except for the annealing temperature at 55°C and for the cycling number of 40.

For *Hepatozoon* detection, the 18S rRNA was targeted by using primers HepF (5′-ATACATGAGCAAAATCTCAAC-3′) and HepR (5′-CTTATTATTCCATGCTGCAG-3′) (26). The reaction contained 12.5 μl of Taq PCR Master Mix (Qiagen, Hilden, Germany), 50 pmol of each primer and 2 μl of DNA, for an overall volume of 25 μl. Thermal cycler conditions were 95°C for 5 min, followed by 35 cycles at 94°C for 1 min, 53°C for 30 s, 72°C for 1 min, and a final elongation step at 72°C for 10 min. Anaplasmataceae were detected with conventional PCR by using primers PER1 (5′-TTTATCGCTATTAGATGAGCCTATG-3′) and PER2 (5′-CTCTACACTAGGAATTCCGCTAT-3′) targeting the 16S rRNA of the bacteria (27). The PCR reaction mixture contained 1X of PCR buffer, 25 pmol of each primer, 0.5 mM of MgCl2, 2.5 U of HotStarTaq DNA Polymerase (Qiagen, Hilden, Germany), 0.2 mM of dNTPs mix (Sigma-Aldrich, St. Louis, MO, USA) and 1 μl of DNA, in a total volume of 25 μl. The thermal cycler conditions were an initial denaturation step at 95°C for 15 min, followed by 40 cycles at 94°C for 1 min, 52.4°C for 45 s, 72°C for 1 min and a final elongation step at 72°C for 10 min.

*Leishmania infantum* was detected by amplifying a fragment of the highly reiterated minicircles of kDNA with primers RV1 (5′-CTTTTCTGGTCCCGCGGGTAGG−3′) and RV2 (5′-CCACCTGGCCTATTTTACACCA−3′) (28). Briefly, the reaction mixture contained 1X of PCR buffer, 22.5 pmol of each primer, 2.5 U of HotStarTaq DNA Polymerase (Qiagen, Hilden, Germany), 0.2 mM of dNTPs mix (Sigma-Aldrich, St. Louis, MO, USA) and 2.5 μl of DNA, in a total volume of 25 μl. Thermal cycler conditions were 95°C for 15 min, 45 cycles of 94°C for 1 min, 62°C for 1.5 min and 72°C for 1 min, and a final elongation step of 72°C for 10 min.

For each PCR, positive and negative controls were processed together with samples and all the precautions were taken to minimize the risk of contamination.

Selected positive amplicons were purified using a commercial kit (Nucleospin Extract II Kit, Macherey-Nagel, Düren, Germany) and sequenced (Macrogen Europe, The Netherland).

Logistic regression was performed by using R software (3.5.1) (29), to investigate possible risk factors for TBD infection (age, sex, area of origin and year of sampling). Map showing the distribution of sampled animals was performed by using GIS (3.2) (30).

## Results

### Prevalence of VBPs

DNA of at least one target pathogen was detected in 93.6% [220/235] of the analyzed animals (Table 2). In particular, 94.9% of the foxes [149/157], 84.8% [28/33] of the wolves and 95.6% [43/45] of the badgers tested positive for any of the analyzed vector-borne pathogen (VBP).

**Table 2:** Prevalence and confidence intervals (CI95%) of the analyzed VBPs divided by species.

**Species**	**Prevalence (Confidence Interval 95%)**			
	***Babesia* spp**.	**Anaplasmataceae**	***Hepatozoon* spp**.	***Leishmania infantum***
Red fox (*Vulpes vulpes*)	89.7% (83.63–93.63%)	10.97% (6.96–16.86)	5.1% (2.62–9.79%)	12.26% (7.99–18.35)
Wolf (*Canis lupus*)	39.4% (24.68–56.32%)	11.43% (4.54–25.95)	75.76% (58.98–87.17%)	25.00% (12.68–43.36%)
European badger (*Meles meles*)	91.1% (79.27–96.49%)	62.22% (47.63–74.89%)	0% (0.00–7.87%)	53.33% (39.08–67.06%)

The prevalence of *Babesia* spp. was significantly higher (*p* < 0.05) in foxes (89.7%, CI95% 83.63–93.63%) [130/145] and badgers (91.1%, CI95% 79.27–96.49%) [41/45] than in wolves (39.4%, CI95% 24.68–56.32%) [13/33], while *Hepatozoon* spp. showed higher prevalence (*p* < 0.05) in wolves (75.76%, CI95% 58.98–87.17%) [25/33] than foxes (5.1%, CI95% 2.62–9.79%) [8/156]. None of the badgers tested positive for *Hepatozoon* spp., although they showed higher prevalence (*p* < 0.05) of Anaplasmataceae infection (62.22%, CI95% 47.63–74.89%) [28/45] and *L. infantum* DNA (53.33%, CI95% 39.08–67.06%) [24/45] than in the other two species (see Table 2).

### Sequencing Results

Results of the sequencing are listed in Table 3. Among the positive samples, 115 amplicons were chosen for sequencing due to the high quality of the PCR products.

**Table 3:** Prevalence, confidence intervals, and identity of each sequenced pathogen divided by host species.

**Pathogen species**	**Host species**	**Sequenced amplicons**	**Prevalence**	**Confidence Interval (95%)**	**Percent Identity**	**GenBank Accession Number**
*B. vulpes*	Fox	15	10.34%	6.37–16.37	99–100%	KM115977
*B. sp DO23163*	Fox	1	0.69%	0.12–3.80	100%	AB935167
*B. capreoli*	Wolf	3	9.09%	3.14–23.57	100%	KX839234
	Badger	1	2.22%	0.39–11.57	100%	KX839234
*B. badger type A*	Badger	7	15.56%	7.75–28.78	100%	KX528553
*B. badger type B*	Badger	1	2.22%	0.39–11.57	100%	KT223485
*A. phagocytophilum*	Badger	3	6.67%	2.29–17.86	100%	KC800985
*Ehrlichia* sp.	Badger	5	11.11%	4.84–23.50	99%	KR262717
*H. canis*	Fox	8	5.13%	2.62–9.79	100%	KU893127
	Wolf	21	63.64%	46.62–77.81	100%	KU893127
*L. infantum*	Fox	19	12.26%	7.99–18.35	100%	HF937257
	Wolf	7	25.71%	14.16–42.07	100%	HF937257
	Badger	24	53.33%	39.08–67.06	100%	HF937257

In foxes, *B. vulpes* was the most prevalent species (10.34%, CI95% 6.37–16.37%), with sequences showing 99–100% of similarity with a sequence described by Duscher and colleagues (31) in hunted foxes from Austria [GenBank: KM115977]. One fox tested positive for *Babesia* sp. DO23163 (0.69%, CI95% 0.12–3.80%), with sequence showing 100% similarity to a sequence obtained from a racoon dog in Osaka, Japan [GenBank: AB935167]. Moreover, 8 foxes tested positive for *H. canis*, and sequences showed 100% similarity with *H. canis* described in hunting dogs from the Czech Republic (32) [GenBank: KU893127]. Three wolves were found to be infected by *B. capreoli*, whose sequence were 100% similar to *B. capreoli* [GenBank: KX839234] identified in horses from Northwestern Italy (33), and 21 by *H. canis*. Sequences of *H. canis* obtained in wolves showed 97–100% similarity to those described in hunting dogs from the Czech Republic (32) [GenBank: KU893127], in Eurasian golden jackals from Austria (34) [KX712123], and in foxes and ticks from Italy (35) [GenBank: GU371448]. Regarding badgers, *Babesia* sp. isolate badger type A was the most prevalent recorded piroplasm (15.56%, CI95% 7.75–28.78%), followed by *Babesia* sp. DO23163 (4.44%, CI95% 1.23–14.83%), *Babesia* sp. isolate badger type B (2.22%, CI95% 0.39–11.57%) and *B. capreoli* (2.22%, CI95% 0.39–11.57%). *Babesia* sp. isolate badger type A sequences showed 100% similarity to a sequence described by Bartley and colleagues (36) in badgers from Scotland [GenBank: KX528553], while the positive samples for *Babesia* sp. DO23163 showed 100% similarity with the sequence we found in one fox in this study. *Babesia* sp. isolate badger type B, found in one badger, was 100% similar to a sequence described by Barandika and colleagues (14) in badgers from Northern Spain [GenBank: KT223485], while *B. capreoli* was 100% similar to *B. capreoli* identified in 3 wolves from this study and horses from Northwestern Italy (33). Finally, *A. phagocytophilum* was detected in 3 badgers out of 8 samples sequenced (6.67%, CI95% 2.29–17.86%), while 5 isolates showed 99% similarity with a novel *Ehrlichia* sp. found in a badger in Northern Spain (37) [GenBank: KR262717]. None of the Anaplasmataceae positive samples obtained from foxes and wolves were sequenced due to the poor quality of the amplicons. Amplicons of *L. infantum* recovered from the animals in this study were sequenced for species confirmation and showed 100% similarity with those deposited (e.g., GenBank:HF937257 from Italy).

Logistic regression showed higher risk of infection for animals collected in flat or hilly areas (below 600 m a.s.l.) than in mountain areas (above 600 m a.s.l.) (AUC = 0.79). In particular, higher risk of Anaplasmataceae infection in foxes (*p* < 0.05; OR = 7.16) and of Babesia sp. in badgers (*p* < 0.01; OR = 22.50) was recorded.

## Discussion

With 23% of emerging infectious diseases actually transmitted by arthropods, vector-borne pathogens have a considerable impact on human health (1). Several factors have been implicated in this emergence; among others, the increase of human encroachment into wild habitats, climate change and the consequent territorial expansion of vector arthropods are the most important (38, 39).

The recent increase of some wildlife populations (38) is expected to influence the epidemiology of vector-borne diseases, as several sylvatic species are known or suspected reservoirs of VBPs.

Few data are available on VBP presence and prevalence in carnivorans from the alpine region (21). In our study, the prevalence of *Babesia* in foxes is one of the highest reported so far in Europe. Similar prevalence has been found in Portugal, where Cardoso and colleagues have reported a prevalence ranging from 78 to 100%, depending on the type of sample (blood or bone marrow) and the analyzed area (northern or southern part of the country) (39). Lower prevalence has been detected in Spain (40), Germany (41), Hungary (42) and Slovakia (43) (72.2, 46.4, 20, and 9.7%, respectively). In Italy, previous findings have showed variable results depending on the geographical area, ranging from <1 to 54% (21–23, 44). Most of the foxes in the present study were found to be infected with *B. vulpes*, as already reported in Spain (45), Italy (46), Croatia (47), Germany (41), Portugal (39), and Austria (31). Despite the severe symptomatology reported in dogs infected with *B. vulpes* (48–50), only one case of symptomatic infection in a fox has been reported so far (51). This finding, together with the high rate of infection reported, may indicate a role of foxes in the sylvatic cycle of this parasite, although more evidences are needed. To date, no proven tick vectors for *B. vulpes* have been observed. The hedgehog tick *Ixodes hexagonus* has been proposed as the main vector of this parasite based solely on the association between the occurrence of this tick and the infection in dogs (52). Moreover, the detection of *B. vulpes* DNA in unfed *Dermacentor reticulatus* ticks in Austria (53) may suggest a possible role of this tick species as well. In the study area, both *I. hexagonus* and *D. reticulatus* have been described infesting privately owned dogs (54).

To the best of our knowledge, only two previous studies have investigated the occurrence of *Babesia* spp. in free-ranging wolves in Europe, reporting a prevalence of 20% in Croatia (10) and 7% in Italy (23). Similar to the present study, Beck and colleagues (10) reported the presence of wolves infected by piroplasms having wild ungulates as natural hosts. In particular, we detected *B. capreoli* DNA in 3 wolf samples, with sequences showing 100% similarity to those reported from sympatric roe deer, red deer, horses and ticks collected from owned dogs (21, 33). Additionally, the same parasite was also found in 1 badger from this study, suggesting a broader host specificity for *B. capreoli* than previously observed. However, due to the limited number of sequenced amplicons, we are not able to speculate any further, and more studies are needed in order to better understand the role of wolves and badgers in the epidemiology of this *Babesia* species. Moreover, most of the badgers and one positive fox were infected with mustelid-related *Babesia* species, such as *Babesia* sp. DO23163, *Babesia* sp. badger type A and type B (13, 14, 36) that belong to the *B. microti* group (13). In Italy, badger-associated *Babesia* infection has been observed also in a wolf from Southern Italy (23), highlighting the circulation of these species within wild carnivorans of the order Caniformia.

*Hepatozoon canis* was the only *Hepatozoon* species detected, with 5% of prevalence in foxes and more than 75% in wolves. Previous reports of *H. canis* in foxes from Italy (35), Croatia (47), Bosnia and Herzegovina (11), and Spain (18, 55) showed a prevalence ranging from 13% up to 90%, while to the best of our knowledge this is the first epidemiological study investigating the occurrence of this parasite in free-ranging wolves. In contrast to *Babesia*, infection with *H. canis* is acquired by the mammal host through the ingestion of an infected tick rather than tick bite. The main vector of this parasite is *Rhipicephalus sanguineus* s.l., the kennel tick, which is widely distributed in Southern Europe and strongly associated with dog presence (56). However, the occurrence of *H. canis* has been reported in wildlife from areas in which *R. sanguineus* s.l. is not endemic such as Austria (57), Slovakia (58), and Germany (59), suggesting the role of other tick species as vectors of *H. canis*. To date, only *Rhipicephalus turanicus* has been considered as an additional definitive host for this parasite (60), while *Dermacentor* spp., *Haemaphysalis concinna* and *Ixodes ricinus* have proved to harbor parasite DNA (35, 61). In Northern Italy, both the proved and the suspected vectors of *H. canis* have been reported in dogs (54) and humans (62). Predation has been proved to be an alternative route for *Hepatozoon americanum* infection, a closely related species endemic in the United States (63), thus suggesting a similar transmission way for *H. canis*. In particular, the consumption of infected carrions and prey such as rodents carrying tissue cysts of the parasite may be a possible infection route for both wild and domestic carnivores (e.g., shepherd and hunting dogs) (61). Finally, transplacental transmission of *H. canis* has been proved in dogs (64) and foxes (65).

Several studies have investigated the prevalence of Anaplasmataceae in wild carnivorans, showing considerable differences among the animal species. In foxes, the occurrence of these bacteria has been previously investigated in Italy (17), Germany (66), Poland (67), The Netherland (68), Romania (69), Switzerland (70), Czech Republic (71), Austria (65), Hungary (16), and Spain (37), with results that are in line with our findings. In contrast to the red fox, badgers and wolves have been less investigated in Europe. No badgers showed positivity for *A. phagocytophilum* in The Netherland (68), Czech Republic (71), and Spain (72), where negative results have been obtained also for the wolf (37). Notably, out of 114 badgers only two were found positive during a study on the occurrence of VBPs in mustelids from Belgium and The Netherland (73). The prevalence of *A. phagocytophilum* obtained in the present study is in line with that reported previously, showing low occurrence of this bacterium in badger and maybe suggesting the poor role of this mustelid species in the epidemiology of Anaplasmataceae. Conversely, additional studies are needed to further investigate the presence of this bacteria in the wolf, due to limited existing information.

More than half of the badgers in our survey tested positive for *L. infantum*, while the prevalence in the other two species is significantly lower (25.71% in wolves and 12.26% in foxes). The occurrence of *L. infantum* has been largely assessed in free-ranging carnivorans, especially in canids, due to their phylogenetic closeness to dogs that are the main reservoir of this parasite (20). The prevalence of *L. infantum* found in foxes in this study, although lower than reported previously in Central and Southern Italy (74, 75), confirms the recently established endemic area of transmission in Northern Italy where autochthonous dogs showed more than 40% of seroprevalence (24). The prevalence in foxes from Spain ranges from 14% (76) to 75% (77), while in Portugal the prevalence is much lower (78, 79). Although several studies have been performed in order to detect *L. infantum* in wolves in Europe (76, 80, 81), to our knowledge this is the first epidemiological study on this parasite in the Italian wolf population. Our results are in line with those obtained from Spain (81), suggesting a similar epidemiological situation. Compared with foxes and wolves, the presence of *L. infantum* in badgers has been generally less evaluated. However, the moderate to high prevalence observed in this and other studies (81, 82) could suggest a role of this species in the epidemiology of *L. infantum*, at least in its sylvatic life cycle. Nevertheless, further studies are needed to assess the capacity of badgers to infect sandflies, a fundamental ability for a competent reservoir (20).

Statistical analysis of risk factors showed a higher risk of Anaplasmataceae infection in foxes and of Babesia spp. in badgers collected in flat or hilly areas (below 600 m a.s.l.) than in mountain areas (above 600 m a.s.l.). This could be accounted to higher abundance of vectors (Ixodid ticks) in hilly areas than in the mountains due to more suitable environmental characteristics and to a higher presence of other sylvatic hosts for ticks as wild ungulates.

## Conclusions

With the molecular analysis of 235 specimens collected from 2009 to 2017, this study provides valuable information about the situation of vector-borne pathogens in wild carnivorans from Northwestern Italy, showing high level of infection in all target species. Moreover, we reported for the first time the presence of *B. capreoli* in wolves and badgers, two unexpected hosts for this parasite, and of *H. canis* in wolves.

This survey highlights the presence of several VBP in the study area, many of which capable to infect domestic animals and humans. The high occurrence of VBPs in sylvatic carnivorans could pose a risk for both animal and human health, especially in an area with growing urbanization and increasing wildlife population as in many parts of Europe, that lead humans, wildlife, livestock and pets to closer contacts.

## Data Availability Statement

All datasets generated and analyzed for this study are included in the article/supplementary material.

## Ethics Statement

Ethical approval was obtained by the Ethical committees of the Department of Veterinary Sciences, University of Turin.

## Author Contributions

EB supervised the data collection, participated in data acquisition, and draft the manuscript. SZ supervised the data collection, undertook statistical analysis, and critically reviewed the manuscript. SK, AT, and BH participated in data acquisition and helped to draft the manuscript. EF conceived the study, coordinated, and supervised the lab force and critically reviewed the manuscript. All authors read and approved the final manuscript.

### Conflict of Interest

The authors declare that the research was conducted in the absence of any commercial or financial relationships that could be construed as a potential conflict of interest.
